# Protocol: Transmission and prevention of influenza in Hutterites: Zoonotic transmission of influenza A: swine & swine workers

**DOI:** 10.1186/1471-2458-9-420

**Published:** 2009-11-18

**Authors:** Margaret L Russell, Julia Keenliside, Richard Webby, Kevin Fonseca, Pam Singh, Lorraine Moss, Mark Loeb

**Affiliations:** 1 Department of Community Health Sciences, University of Calgary, 3330 Hospital Drive NW, Calgary, Alberta T2N 4N1, Canada; 2 Food Safety and Animal Health Division, Alberta Agriculture and Rural Development, 6909 - 116 St, Edmonton, Alberta T6H 4P2, Canada; 3 Department of Infectious Diseases, World Health Organization Collaborating Center for Studies on the Ecology of Influenza in Animals and Birds, St Jude Children's Research Hospital 262 Danny Thomas Place Memphis, TN 38105, USA; 4 Provincial Laboratory for Public Health (Microbiology) 3030 Hospital Dr NW Calgary, Alberta T2N 4W4, Canada; 5 Department of Pathology & Molecular Medicine, McMaster University, Room 3200, Michael G DeGroote Centre for Learning and Discovery, 1200 Main St W Hamilton, ON L8N 3Z5, Canada; 6 Departments of Pathology & Molecular Medicine, Medicine, Epidemiology and Biostatistics, Michael G DeGroote Institute for Infectious Disease Research Room 3203 Michael G DeGroote Centre for Learning and Discovery McMaster University, 1200 Main St W Hamilton, ON L8N 3Z5, Canada

## Abstract

**Background:**

Among swine, reassortment of influenza virus genes from birds, pigs, and humans could generate influenza viruses with pandemic potential. Humans with acute infection might also be a source of infection for swine production units. This article describes the study design and methods being used to assess influenza A transmission between swine workers and pigs. We hypothesize that transmission of swine influenza viruses to humans, transmission of human influenza viruses to swine, and reassortment of human and swine influenza A viruses is occurring. The project is part of a Team Grant; all Team Grant studies include active surveillance for influenza among Hutterite swine farmers in Alberta, Canada. This project also includes non-Hutterite swine farms that are experiencing swine respiratory illness.

**Methods/Design:**

Nurses conduct active surveillance for influenza-like-illness (ILI), visiting participating communally owned and operated Hutterite swine farms twice weekly. Nasopharyngeal swabs and acute and convalescent sera are obtained from persons with any two such symptoms. Swabs are tested for influenza A and B by a real time RT-PCR (reverse transcriptase polymerase chain reaction) at the Alberta Provincial Laboratory for Public Health (ProvLab). Test-positive participants are advised that they have influenza. The occurrence of test-positive swine workers triggers sampling (swabbing, acute and convalescent serology) of the swine herd by veterinarians. Specimens obtained from swine are couriered to St. Jude Children's Research Hospital, Memphis, TN for testing. Veterinarians and herd owners are notified if animal specimens are test-positive for influenza. If swine ILI occurs, veterinarians obtain samples from the pigs; test-positives from the animals trigger nurses to obtain specimens (swabbing, acute and convalescent serology) from the swine workers. ProvLab cultures influenza virus from human specimens, freezes these cultures and human sera, and ships them to St. Jude where sera will be examined for antibodies to swine and human influenza virus strains or reassortants. Full length sequencing of all eight genes from the human and swine influenza isolates will be performed so that detailed comparisons can be performed between them.

**Discussion:**

The declaration of pandemic influenza in June 2009, caused by a novel H1N1 virus that includes avian, swine and human genes, highlights the importance of investigations of human/swine influenza transmission.

## Background

The reassortment of genes from influenza viruses from different animal species circulating within pigs is thought to be one mechanism for the development of influenza viruses with pandemic potential [1]. Although the host range restriction of influenza viruses is a polygenic trait, the haemagglutinin (HA) glycoprotein is critical as it is responsible for viral attachment to the sialic acid receptors on the host cell surface. While human and avian viruses differ in the nature of the sialic acid receptors they prefer, both avian and human influenza viruses can infect pigs because porcine cells in the respiratory tract express the linkages for both human and avian strains [2]. In both the United States and Canada since 1998, triple reassortant H3N2 influenza viruses containing human, classical swine, and avian virus lineage genes have been isolated from pigs [3,4]. In Canada, both wholly human H1N2 and human-swine reassortant H1N2 viruses have been recovered from pigs from the province of Ontario [3]. A reassortant H1N1 virus of a unique genotype not previously seen in pigs that contained genes of classical swine virus lineages as well as a polymerase gene of a human virus lineage polymerase was also found, suggesting that co-infection and reassortment are occurring [3,5]. Transmission of influenza between humans and swine is known to occur, although the frequency of such occurrence is not well understood [6]. There may also be significant economic and production consequences to influenza virus infection in naïve commercial swine herds; therefore cross species infection is also of concern to veterinarians and agricultural producers from an economic and animal health perspective. A better understanding of the transmission of influenza between humans and swine is thus important, and will have implications for pandemic preparedness, particularly in light of the recently declared influenza A (H1N1) 2009 pandemic [7]. An ideal population in which to study this would be one in which humans, swine and poultry exist in close proximity. To this end, we describe a unique Canadian model, a study of influenza transmission on Hutterite colonies.

### Why Hutterites?

The Hutterites live on communally owned and operated farms (colonies). These colonies commonly raise swine and are relatively isolated from towns and cities. This combination of isolation from the larger Canadian population plus intensive within-colony social contact may facilitate influenza transmission within the colony, while relatively reducing re-introduction of the virus from the wider community. In contrast to most other swine farmers, Hutterite colonies usually also raise poultry and domestic waterfowl, are located under major wildfowl flyways, and their lands include ponds and dugouts that are attractive to wildfowl and are frequently used as a source of water for the colony livestock operations. This makes Hutterite farms uniquely desirable sites for studying interspecies transmission of influenza viruses, including detection of subtypes not commonly seen in humans (H4, H5, H7, H9), but potentially circulating among poultry and domestic and wild waterfowl [8]. In 2006, a Hutterite child in Alberta was hospitalized with respiratory symptoms that were subsequently shown to be due to influenza A [9]. This was a novel H3N2 strain closely related to a human/classic swine/avian triple reassortant strain previously isolated in Ontario [5,9]. This suggested to us that there would be interest in the Hutterite community in participating in a study of influenza transmission that included both people and pigs.

### The Hutterites

Hutterites are members of a Protestant sect founded as part of the 16^th ^century Anabaptist movement. In the nineteenth century, they immigrated as a group to Canada and the United States [10,11]; where at the present time some three quarters of the colonies are in Canada. In Canada, there are 30,665 Hutterites [12] living on 341 colonies [13]. Nearly all Canadian Hutterites (98.5%) live in the Canadian prairie provinces (Alberta 48.9%, Manitoba 30.7%, Saskatchewan 18.8%) [12]. Eight to 12 families comprise the estimated 90 - 100 persons who live on each colony [13-15]. Colonies have their own one-room schools.

Although property is communally owned, colony members live within nuclear families, with each family living within a separate unit of a 'row house' [15,16]. Meals are taken communally in a central dining hall, and men, women and children eat separately [17,18]. Gender roles and the hierarchy on Hutterite colonies are thought to be divinely ordained; older people have authority over younger and men over women [19]. Men and women engage in separate work, with women being limited to "family, domestic and food preparation jobs" [10]; while men engage in farm production and farm operations [10,20]. Schooling for Hutterite children generally ends at age 15 years [18] and young men serve as apprentices for about two years, acting as a mobile labour force within the colony [10].

The gender distribution among Hutterites is similar to that of other Canadians [21,22]; however, the Hutterite population is younger: 5.1% aged 65 years or older in contrast to 13.0% of the Canadian population, or 10.4% of the Alberta population [21,22]. The Hutterite population also has a higher fertility rate than Canadians generally. In recent years the fertility rate for Canadians, generally, has ranged between 1.51 and 1.54 children per woman [23]. However, although declining in recent years, the corresponding rate for Hutterite women is 6.29, and the average family size in the order of 5 [20,24].

Hutterites are active swine farmers, owning more than one third of all of the pigs in Alberta and Saskatchewan [25] and about 40% of those in Manitoba [26]. The farrow-to-finish hog production system (i.e., all phases of hog production from breeding to growing the pigs to market weight) is frequently used. A typical operation has 300 - 600 sows and produces 6,000 - 13,000 pigs annually [25]. Hutterite farmers are efficient and produce a disproportionately large amount of agricultural produce [27,28]. On a typical colony there will be a pig-boss [10], and there may be 2 - 10 workers (including mobile "apprentices") who work in the hog barns (J. Keenliside, personal communication, July 2007).

## Study Purpose and Objectives

The 'Flu Zoonotic' study (Zoonotic transmission of Influenza A: swine & swine workers) is one of six studies that comprise a Team Grant funded by the Canadian Institutes of Health Research (Program: Pandemic Preparedness - Influenza Transmission and Prevention). It is conducted in the province of Alberta. The major goals of the Team Grant are to:

• Develop a better understanding of the natural history of influenza

• Investigate determinants of successful influenza immunization, and to

• Gain a better understanding of influenza transmission and its prevention in the community.

The objective of the "Flu Zoonotic" study is to assess influenza A transmission between swine workers and pigs. We will determine whether serological evidence of influenza A virus exists in either swine or humans. We will assess if swine to human/human to swine transmission of influenza A (human virus, swine virus, reassortants) is occurring and will characterize the swine influenza viruses that infect humans and swine. We hypothesize that transmission of swine influenza viruses (SwIV) to humans, transmission of human influenza viruses (HuIV) to swine, and that reassortment of human and swine influenza A viruses is occurring.

## Methods/Design

This is a prospective cohort study conducted on Hutterite colonies ("surveillance farms") in Alberta, Canada, where ongoing surveillance for influenza is conducted among humans and their swine. The occurrence of illness among humans triggers swine testing, similarly, the occurrence of illness among swine triggers human testing.

### Surveillance farms

Each fall baseline sera and information is collected on human participants and on the swine herd. A nurse administered structured interview is used to collect baseline information from participating colony members, including age, medical history, influenza immunization including receipt of the 1976 swine flu vaccine, and previous and current exposure to swine. A veterinarian obtains a standardized herd history from the pig boss, including a descriptor of the type of operation (e.g., farrow-to-finish, farrow-to-wean, grow/finish etc.), closed vs. open herd, number of sources from which pigs are introduced (for open herds); total herd size and size by herd segment (sows, gilts, growers, finishers, nursery pigs/weaners), swine vaccination history, history of prior influenza in herd; history of other swine respiratory diseases in herd, whether or not poultry or water fowl are raised on the farm, and a detailed checklist of on-farm biosecurity practices.

The surveillance period for this study is defined on the basis of data from the Alberta wide human influenza surveillance program [29], focusing on sentinel sites that are in the geographic area that includes the participating Hutterite colonies. It begins when ≥1 nasopharygneal or throat swab specimens received by ProvLab are positive for influenza A and B viruses for two consecutive weeks. Surveillance stops once there have been two consecutive weeks of no positive influenza A or B patients detected in these areas. Nurses visit each participating colony twice weekly during the surveillance period and take nasopharyngeal swabs (placed in Universal Transport Medium - UTM) plus acute and convalescent blood samples from those who have experienced 2 or more symptoms from a close ended checklist (fever [≥ 38^0 ^Celsius], cough, nasal congestion, sore throat, headache, sinus problems, muscle aches, fatigue, ear ache or infection, chills). Among those classified as swine workers (at least one hour per week spent in swine barns), a detailed history of exposure to poultry and birds and related work practices including on farm biosecurity practices is obtained if any polymerase chain reaction (PCR) test is positive for influenza A. If two or more swine workers on the colony have symptoms within a 72 hour period, nasopharyngeal swabs (flock nasopharyngeal and flock nasal swabs) and blood specimens (acute and convalescent) from all swine workers on the colony are obtained. The respiratory samples are immediately tested by real time RT-PCR for influenza A and B virus and the virus is also cultured from positive samples at ProvLab. If any samples are test positive, specimens are collected from a sample of pigs from the same colony. In the event that influenza is identified in the pigs, the study nurse administers a questionnaire and collects specimens (flocked nasal and nasopharyngeal swab for influenza A, acute and convalescent blood samples taken 3 weeks apart) from all of the swine workers on the affected colony, whether or not they are symptomatic.

At each twice weekly colony visit, nurses also inquire of the pig bosses if there has been any swine illness. Herd veterinarians also look for evidence of influenza during their routine barn visits and telephone consultations with the pig boss. If there has been swine illness, the nurse or veterinarian notifies an investigator (JK or MLR) who alerts a study veterinarian to investigate the illness. If the study veterinarian suspects swine respiratory illness/swine influenza, based on symptoms of one or more of: fever, sneezing, cough, huddling, loss of appetite (decreased feed consumption), stunted growth (i.e., pigs in the same pen visibly vary in size and shape) or increased mortality with or without cough; he/she makes a site visit to the farm. The veterinarian takes samples from 30 pigs including those that are most overtly ill, sampling from each age group. Samples include Dacron^® ^nasal swabs and 2 blood samples (3 weeks apart) for acute and convalescent serology from at least 24 pigs and lung tissue from up to 6 pigs. This tissue is obtained from on-farm post-mortem after the pigs have been purchased from the farmer and euthanized. In the event of illness in a pig worker and the absence of clinical disease in swine, only nasal swabs and blood samples for acute and convalescent serology are collected from 30 pigs. Swabs are placed in standard virus transport medium. Lung samples are to be collected from each pig, divided in half; placing one half in a tube containing RNAlater^® ^(Ambion) medium and the other half (for PCR testing and virus culture) in virus transport medium. Specimens are immediately placed in field coolers with appropriate gel packs for transport.

### Outbreak farms

This component is done 12 months of the year and best considered as a "snap-shot". It was added to the protocol in 2008 when additional funding (Alberta Livestock and Meat Agency) became available to increase the number of herds from which samples could be obtained, thus increasing the likelihood of isolating the viruses of interest. Community veterinarians, when called to attend swine illness on farms that are not enrolled in the surveillance cohort (regardless of whether or not the farmers are Hutterites), inform the producers of our study and invite them to participate. If the producer consents, the veterinarian obtains the standardized herd history and collects samples as per the occurrence of swine illness on surveillance farms. A study nurse is called and obtains consent to participation from farm workers. A history is obtained and nasopharyngeal swabs plus blood samples taken (3 weeks apart) for acute and convalescent serology.

### Veterinarians and farm visits

The veterinarians for this study include one investigator and community veterinarians within the geographic study area. Whenever possible these are the veterinarians who usually provide services to the participating farms. Many have specialized swine practices. We chose as much as possible to use those veterinarians with whom the farmers had already established relationships, to build trust with both farmers and the local veterinary community. For those farms that do not have a usual attending veterinarian, we have a listing of participating community veterinarians within the geographic area from which farmers may select a practitioner. All veterinarians respect farm-specific biosecurity requirements, including minimum intervals specified by farmer since visits to other swine barns.

### Specimen transport

Human swabs and sera are sent to ProvLab using the usual regional health authority transportation system for human specimens. Swabs are tested for influenza A and B using real time RT-PCR testing. The nurses are told by telephone if the sample is positive for influenza, and in turn notify positive participants that they have influenza. Virus is cultured and frozen, and the cultures and sera transhipped to St. Jude Children's Research Hospital (Memphis, TN) for batch analysis.

The transportation system for animal specimens was developed specifically for this study and pilot tested in 2007 - 2008. Although the use of viral transport medium should ensure that virus will maintain viability under refrigeration, we pre-tested the effect of transportation procedures on virus viability by using a sample of viable human influenza virus in virus transport medium (provided by ProvLab) in 2 pre-tests (including courier to Memphis and culture in Memphis).

Animal specimens are shipped by overnight courier to the World Health Organization Collaborating Center for Studies on the Ecology of Influenza in Animals and Birds (St Jude). Swabs and lung tissue are tested for influenza using real time RT-PCR and the veterinarians and pig owners/pig bosses informed if tests are positive for influenza.

### Laboratory analyses

At St. Jude, animal specimens that are positive for influenza A by real time RT-PCR are cultured on Madin Darby canine kidney cells and in 10-day-old embryonated chickens' eggs according to the published guidelines of the World Health Organization [30]. Nucleotide sequences of the full-length coding regions of all 8 RNA segments from each virus will be determined by direct cycle sequencing with previously described techniques and primers [31-33]. Human sera (batched and frozen at -80 degrees Celsius and shipped annually to Memphis) and pig sera will be tested for antibodies to the anticipated human influenza A strain for the season such as, for the 2007-2008 season, A/Solomon Islands/3/2006 (H1N1) and A/Wisconsin/67/2005 (H3N2); and for the swine strains that are known to be recently circulating in Canada [e.g. A/Swine/Ontario/33853/05 (H3N2) - like; A/Swine/N Carolina/18161/02 (classical H1N1 - like)] as well as to a more recent isolate with human-like H1 gene, A/Swine/North Carolina/24848-1/05). Microneutralization assay will be used to test human and swine serum samples for antibodies to H4, H5, H7, and H9 influenza viruses due to the assays' increased sensitivities and specificity over hemagglutination inhibition assays [32] according to the WHO recommended procedures [30]. The specific antigens to be used will be selected from ongoing virologic surveillance in duck populations in Alberta [33].

Serological evidence of transmission of SwIV/reassortants to swine workers will be defined as antibodies to one or more of the swine strains or reassortants with a titre > 1/40 [34]. This is valid only for classical swine H1N1 virus in human sera collected before April 2009, as, due to shared epitopes, there may be cross reactivity on serological tests between endemic human and swine strains, including the new pandemic influenza strain. Evidence for transmission of other swine strains/reassortants will require culture from the swine workers. Serological evidence of transmission of HuIV/reassortants to pigs is strongest if there is an eightfold or higher increase in titre between acute and convalescent serum samples to contemporary human H3N2 and H1N1 viruses. Further evidence of transmission from human to pig is the isolation of these human strains from pigs, the strongest evidence obtained from comparison of the full length sequences showing that the same strains are isolated from both humans and animals.

### Ethics

We do not do virus subtyping or sequencing or serological analyses in real time. All samples are shipped to laboratories under code and all testing done in an anonymized fashion to preclude linkage of results to a specific farm and is done at periodic intervals such that evidence of infection would be historical (i.e., sufficient time would have passed since specimen collection that any infection events would be over, precluding the need for any public health action). This is required to abate concerns that study herds would be quarantined or depopulated or the market value of the swine from the colony or the Alberta swine industry, generally, be adversely impacted by test results. This study was approved by the Conjoint Health Research Ethics Board of the University of Calgary (Ethics ID 18970), McMaster University HHS/FHS Research Ethics Board (REB project # 07-376), and the University of Calgary Animal Care Committee (Protocol M07107).

## Discussion

The circulating H1N1 2009 pandemic strain of influenza is thought to have reassorted within swine [35]. The first isolation of the pandemic strain from swine occurred on an Alberta farm [36] that had been targeted for inclusion as an "outbreak farm" under this study protocol. The infection of the pigs on this farm and farms elsewhere by the pandemic strain is thought to be the result of human to pig transmission [37]. This highlights the importance of this study and of global surveillance of influenza transmission between humans and pigs.

## Competing interests

The authors declare that they have no competing interests.

## Authors' contributions

MLR conceived of the study, participated in its design and coordination and drafted the manuscript. JK participated in study design and coordination, contributed to data acquisition and helped to draft the manuscript. RW participated in study design, will do the virological analysis and helped to draft the manuscript. ML participated in study conceptualization and design and coordination and helped to draft the manuscript. KF participated in study design, contributed to data acquisition, will contribute to the virological analysis and helped to draft the manuscript. PS participated in study coordination and helped to draft the manuscript. LM participated in study coordination and helped to draft the manuscript. All authors participated in revising the manuscript critically for intellectual content. All authors have given final approval of the version to be published.

## Pre-publication history

The pre-publication history for this paper can be accessed here:

http://www.biomedcentral.com/1471-2458/9/420/prepub
